# Depression in the menopause transition: risks in the changing hormone milieu as observed in the general population

**DOI:** 10.1186/s40695-015-0002-y

**Published:** 2015-08-11

**Authors:** Ellen W. Freeman

**Affiliations:** Department of Obstetrics/Gynecology and Department of Psychiatry, Perelman School of Medicine, University of Pennsylvania, 3701 Market Street, Suite 820 (Mudd Suite), Philadelphia, PA 19104 USA

**Keywords:** Depression, Menopause, Transition to menopause, Menopausal symptoms, Perimenopause, Reproductive hormones, Estradiol

## Abstract

There is accumulating evidence but no definitive answers about the incidence of depressed mood in the menopause transition and its association with the changing hormonal milieu. While a changing hormonal milieu is the natural condition for all women, only a minority of mid-life women experience debilitating depressive symptoms or clinical depression. This review focuses on associations between depressed mood and the menopause transition, primarily as identified in longitudinal, population-based studies in the past decade. Further aims were to present reported associations between depressed mood and reproductive hormones in the menopause transition as evaluated in the general population and associations of depressive symptoms or clinical depression with menopausal hot flashes or poor sleep in perimenopausal women. There is evidence to support the role of the changing endocrine milieu in the development of depressed mood in the menopause transition, but the contribution of hormones as measured is small. Disentangling the numerous factors that are associated with depression in midlife women is a major challenge for research and for clinical care, where treatments are needed to improve the most distressing menopausal symptoms.

## Introduction

Changes in menstrual bleeding patterns signal the approach of menopause in mid-life women, and many women report hot flashes, poor sleep, depressed mood and other symptoms along with these menstrual changes. The extent to which symptoms that arise around menopause are directly associated with the hormone changes of ovarian aging is not well understood. There is accumulating evidence but no definitive answers about many potential risk factors and the role of the changing hormone milieu associated with depressed mood around menopause. Further information is clinically important, because of the diminished functioning and significant disability that accompany depression [1, 2], the exacerbation of disorders associated with the neuroendocrine system [3], and the associations of depression with other major health problems, including cardiovascular disease [4–6], metabolic syndrome [7–9] and osteoporosis [10, 11], that complicate treatments and contribute substantially to disability and the high costs of health care.

Depressive symptoms are common in all populations but appear to increase among women in the transition to menopause. Major depression is more common in women than in men in all age groups until late life, with a lifetime prevalence of 21 % compared to 12 % for men in the National Comorbidity Survey [12]. Depression in women also appears to increase around reproductive events. Postpartum depression following childbirth, premenstrual dysphoric disorder linked to the menstrual cycle, and depression around menopause may possibly share a sensitivity to normal shifts in reproductive hormones, which in turn modulate neuroregulatory systems associated with mood and behavior [13, 14]. However, definitive answers about these associations, the etiology of depression, and the role of reproductive hormone changes in depressive disorders have been elusive.

Reports pertaining to depression around menopause have changed over the years. In 2005, the National Institutes of Health (NIH) issued a State-of-Science review of the management of menopause-related symptoms, which concluded that evidence for associations between depressed mood and menopausal status was poor or mixed [15]. Another review of prospective cohort studies that assessed mood symptoms of mid-life women around menopause similarly concluded that there was no clear evidence of an independent effect of the menopause transition on depression [16]. These conclusions stimulated further research leading to more precise definitions of menopausal status and the use of standard measures of depressive symptoms or depressive disorders. Subsequently, multiple, but not all, population-based studies reported an increased prevalence of depressive symptoms in the menopause transition compared to premenopausal women [17]. Some studies differentiated a first-onset depression from a history of depression in perimenopausal women. The studies overall showed that both women with a history of depression and women with no history of depression had an increased risk of depressive symptoms and possibly an increased risk of depressive illness around menopause, but the prevalence was significantly greater among women with a history of depression [18–20].

The aims of this review were to examine associations between depressed mood and the menopause transition, focusing on longitudinal studies in the general population. Further aims were to present reported associations of reproductive hormones with depression in the menopause transition, observed associations between depressive symptoms and menopausal hot flashes or poor sleep, which have been identified as risk factors for depressive symptoms or clinical depression in perimenopausal women. The review did not include intervention studies, neuroendocrine studies, animal models, or studies of postmenopausal women. These domains are clearly important for understanding the development of depression around menopause but are beyond the range of this review and are examined elsewhere [14, 21–29].

## Review

### Common menopausal symptoms

More than 80 % of women experience physical or psychological symptoms around menopause, with varying degrees of severity and disruption in their lives [30, 31]. Depression and other mood changes, hot flashes and sleep difficulties are among the most frequently reported menopausal symptoms [32, 33], although reports of their prevalence and duration can vary widely, due in part to differences in symptom measures, definitions of menopausal status, retrospective or prospective observations in relation to menopause, cross-sectional study designs, sample sizes, and adjustments for confounding factors.

### The menopause transition (perimenopause)

Menopause is defined post hoc after no menstrual bleeding for at least 12 months [34]. In the classic longitudinal study of Treloar, the mean age at menopause was 50.7 years, with a 95 % range of 44–56 years [34]. In the Massachusetts Health Study of the normal menopause transition, the *median* age at menopause was 51.3 years [35]. The transition to menopause is marked by progressively expanding variability in menstrual bleeding, which results from the increasing frequency of anovulation as the number of oocytes declines. The data of Treloar show that this reproductive transition extends for 2 to 8 years before menopause, and that the age at onset of the menopause transition ranges from 39 to 51 years for 95 % of women [34]. Thus, the mean duration of the menopause transition is about 5 years, although both the age at onset and the duration vary widely among individuals, and factors such as smoking, obesity and mood disorders may alter the timing or the duration of the transition period [36–39].

The development of a staging system for reproductive aging by the Stages of Reproductive Aging Workshops (STRAW) led to considerable increases in information about the rise of symptoms in relation to menstrual status and potential associations between symptoms and hormone changes of ovarian aging. The STRAW definitions of stages of reproductive aging were based primarily on observed bleeding patterns. The definitions were further refined as data accumulated, with revised definitions presented in STRAW + 10 [40]. The STRAW-defined stages in the menopause transition are early transition (persistent difference of 7 days or more in the length of consecutive menstrual cycle) and late transition (amenorrhea of 60 days or longer). Postmenopause is divided into early (2 years: +1a, +1b; and 3–6 years: +1c after the final menstrual period) and late (the remaining lifespan) stages. Perimenopause indicates the time around menopause and extends from the early transition stage through 12 months after the final menstrual period [40].

### Prevalence of depression

The National Comorbidity Study reported 30- day estimates of major depression for women aged 45–54 years of 5.0 % and lifetime estimates of 21.8 % [41]. Depression tends to wax and wane with repeated episodes or persist in a chronic state, which occurs in up to 35 % of depressed patients as reported by Nierenberg [42]; a prior depression is the strongest predictor of a subsequent depression [43]. The rates of *recurrent* depression for women are highest in the 45–54 year age-group [41], which are the years proximal to menopause. Consequently, recurrent depression may coincide with the perimenopausal years, contributing to reports of increased prevalence of depression around menopause.

### Depression and the menopause transition

An association between depression and menopausal status is one of the most controversial issues in the menopause transition. Epidemiologic studies based on self-report of menopausal status and depressed mood consistently indicated that most respondents did not report high rates of depressive symptoms, and that reported depressive symptoms were not associated with menopause per se but with other health problems [16, 43–46]. Bosworth et al. reported that 28.9 % of women aged 45–54 years had a high level of depressive symptoms based on an abbreviated CES-D scale but found no association between the depressive symptoms and menopausal status as defined by the women’s perceptions [47]. In the Massachusetts Women’s Health Study, there was an increased risk of depression in the menopause transition but no significant association between the increased risk of depression (CES-D scores) and the onset of menopause [43]. A subgroup of women with a long transition period had an increased risk of depression, but the causal direction could not be determined; the researchers speculated that the increased risk could be explained by increased menopausal symptoms.

Early data from the large Study of Women’s Health Across the Nation (SWAN) indicated that rates of persistent mood symptoms were higher in early perimenopausal women than among premenopausal women (18.4 versus 14.9 and 12 % versus 8 %, respectively) [48]. Dennerstein et al. defined perimenopause status from bleeding patterns and reported that more late perimenopausal women reported depressive symptoms in the previous 2 weeks (38 %) compared to premenopausal (26 %) and postmenopausal women (28 %) [49]. In contrast to community-based samples, evaluation of women attending a menopause clinic identified higher rates of depressive disorders. Soares et al. reported that 28.7 % of women ages 40–58 years in a menopause clinic met DSM-IV criteria for depressive disorders [50]. These studies all suggested an increased prevalence of depressive symptoms and possibly depressive illness in the transition to menopause.

As studies more specifically defined a menopause transition period and systematically assessed depression status, associations between depressive symptoms and menopausal status were consistently observed in longitudinal cohort studies.

The Harvard Study of Moods and cycles found that women with no history of depression were nearly twice as likely to experience depression in the menopause transition compared with premenopausal women (OR 1.8, 95 % CI:1.0 – 3.2) [36]. When adjusted for a history of adverse life events and vasomotor symptoms, the risk of depressive symptoms further increased to 2.5 times for women in the menopause transition compared with premenopausal women (OR 2.5; 95 % CI: 1.2 – 5.2).

In a series of studies in the Penn Ovarian Aging cohort (POAS), the risk of depressive symptoms (CES-D scores) was nearly three times greater in women in the menopausal transition compared with premenopausal women, after adjusting for other predictors of depression including a history of depression, hot flashes, poor sleep, and severe premenstrual syndrome (early transition OR 1.55, 95 % CI: 1.04 – 2.32 and late transition OR 2.89, 95 % CI: 1.29 – 6.45) [51]. In a further study of women with no history of depression, the risk of CES-D depression was more than four times greater in the menopause transition compared with when the same women were premenopausal (OR 5.44; 95 % CI: 2.56 – 11.59 in adjusted analysis) [19]. When only women who had a DSM-IV clinical diagnosis of depression were evaluated, the odds of the onset of a diagnosed depressive disorder were significantly greater in the menopause transition compared with the woman’s own premenopausal period (OR 1.60; 95 % CI: 1.25 – 5.02 in adjusted analysis) [19]. When depressive symptoms (high CES-D) were evaluated across 14 years of follow-up relative to the final menstrual period (FMP), the findings demonstrated a pivotal role of the FMP in the risk of depressive symptoms: there was a higher risk of high depressive symptoms before and a lower risk after the FMP [18].

A series of SWAN studies evaluated menopausal status in relation to outcomes of dysphoric mood [48, 52], depressive symptoms (CES-D) [53–55] and depressive disorder [56, 57]. These studies overall showed that depressive symptoms and dysphoric mood varied by menopausal status and were independent of other known risk factors for depression. Women were more likely to report depressed mood (CES-D scores) in the menopause transition and early postmenopausal years compared with premenopause after adjusting for other demographic psychosocial, behavioral, and health factors [54]. The likelihood of depressive symptoms was significantly higher in the early transition (OR 1.30; 95 % CI: 1.09 – 1.55), the late transition (OR 1.71; 95 % CI:1.27 – 2.30), and postmenopause (OR1.57; 95 % CI: 1.15 – 2.15) compared with premenopausal women [54]. Perimenopausal women were nearly twice as likely to experience a major depressive episode after adjusting for a history of depression, vasomotor symptoms, BMI, age, race, upsetting life events and medication use (OR 1.98; 95 % CI: 1.00 – 3.92) [56]. The likelihood of experiencing an episode of major depression during a 10 year follow-up did not differ between African American and Caucasian women [56]. When African American women were hypothesized to have greater persistence or recurrence of mood disorders compared to Caucasian women, no significant difference in recurrence of depression was found between African American and Caucasian women in 11 years of follow-up [58].

The Seattle Midlife Women’s Health Study (SMWHS) showed that depressive symptoms (CES-D scores) significantly increased in the late menopause transition stage after adjusting for multiple risk factors (beta 1.56, SE 0.73, *P* = 0.032) [59]. Menopausal stage increased depression scores more than age and the late transition stage remained an independent factor after adjustment for age, life stress, body mass index, history of postpartum blues, and use of antidepressants, each of which remained significantly associated with depressed mood in the final adjusted model.

In the Melbourne study, depressive symptoms (a shortened version of CES-D) were higher in women who were in the early and late stages of the menopause transition compared to postmenopausal women [60]. CES-D correlated with negative mood measured concurrently with Affectometer (*r* = 0.63) and baseline negative mood (*r* = 0.37). Prior negative mood, history of premenstrual complaints, negative attitudes toward aging or menopause, poor health, and daily hassles significantly predicted depressed mood.

The Norway HUNT-II study added further support to evidence of increased depressive symptoms in the menopause transition. Based on the Hospital Anxiety and Depression Scale (HADS), a widely-published, brief self-report measure that is validated to detect the presence and severity of depression and anxiety in non-psychiatric populations) [61], scores for depression and anxiety were significantly higher for perimenopausal women compared to premenopausal women [62].

Several studies evaluated the risk of *clinical depression* in the menopause transition but had conflicting results. In a study of 29 women who met clinical criteria for depression, the risk of a depression onset was significantly greater in the 2 years surrounding the final menstrual period than in the preceding premenopausal years [63]. Two population-based studies extended these findings [19, 36], although conclusions are limited by different definitions of new onset depression in these studies.

A third community-based study utilized a standard psychiatric interview to identify current major depression over a 7-year time period; 42 cases of first-onset major depression were identified, but no significant associations were found between first-onset major depression and menopausal status or reproductive hormone levels [57]. The identification of major depression is a strength of the study. However, the small number of identified cases may have yielded less reliable estimates, and interpretation of the results must consider the possibility of type 11 error.

The Harvard Study of Moods and Cycles enrolled a second cohort to continue studies of new-onset depression in late reproductive-age women. Unexpectedly, the rates of new-onset depression were extremely low in the second cohort [64]. The risk of incident major depression or dysthymia over a 3-year period was 5.7 % in cohort 1, but only 1.2 % in cohort 2, yielding a highly significant difference (4.5 %, 95 % CI: 2.2, 6.8). An in-depth analysis to determine possible sources of bias for these disparate findings identified selection bias associated with self-referral of the participants and elements of the study design as the likely reasons for the very disparate rates of depression identified in the two cohorts. These findings underscore the importance of considering the possible impact of selection biases, particularly in populations with differing racial and economic conditions.

In summary, these longitudinal studies in the general population consistently showed that depressive symptoms were greater in the menopause transition compared to premenopause. However, the use of standard diagnostic criteria for *depressive illness* was limited, and evidence for an increased risk of diagnosed depressive illness (rather than depressive symptoms) in the menopause transition was less clear. This was emphasized in another review of relationships between menopause and depression, where the researchers concurred that “the change in menopausal status over time was associated with an increased risk of elevated depressive symptoms, independent of demographic, psychosocial, behavioral and health factors” [65]. However, when menopause-associated depressions were distinguished from depressive disorders, the more limited data from women with depressive disorders were insufficient to conclude that depressive illnesses occurred as a biological response to hormonal changes in the menopause transition.

### Hormone associations with depression in the menopause transition

Associations of reproductive hormones with mood are difficult to measure in large epidemiologic studies. Differences in frequency and timing of measurements, assay sensitivities and different statistical models in analysis of the data contribute to the limited and inconsistent findings of hormone associations with depression in the menopause transition.

Accumulating data suggest that perimenopausal depression is not simply due to low hormone levels, but that fluctuations or changes in hormone levels, which characterize the transition to menopause, may be endocrine triggers for perimenopausal depression is some women [14]. Evidence from clinical research and animal models was reviewed by Gordon et al. as a basis for a theoretical model for perimenopausal depression [21]. In the conceptual model, ovarian hormone fluctuations, particularly progesterone-derived neurosteroids, were modulators of the GABA-ergic modulation of the hypothalamic-pituitary-adrenal (HPA) axis. This may sensitize vulnerable women to psychosocial stress and potentially lead to increased vulnerability to depression in some women. The researchers concluded that “this proposed model provided a basis for understanding the mechanisms by which the changing hormonal environment of the menopause transition may interact with the psychosocial environment of midlife to contribute to risk of perimenopausal depression”.

Another review considered the role of allopregnanolone, a stress-responsive regulator of neuronal function, in triggering affective dysregulation in susceptible women [66]. They concluded that the source of susceptibility to affective disorder remains unclear, but evidence in multiple animal models suggests that allopregnanolone may trigger mood symptoms, possibly as a function of alterations in central neurotransmitter systems, possibly y-aminobutyric acid (GABA), consequent to allopregnanolone withdrawal combined with stress.

Several epidemiologic studies have evaluated hormone variability in relation to mood in the menopause transition with conflicting results. In an 8-year follow-up of the SWAN cohort, testosterone levels and increased testosterone from baseline were associated with an increased likelihood of high depressive symptoms (CES-D scores) (OR 1.15; 95 % CI: 1.01 – 1.31 and OR 1.23; 95 % CI: 1.04 – 1.45, respectively) [55]. There were no associations with depressive symptoms of either levels or changes in estradiol or FSH. A subsequent study of the SWAN mental health sample evaluated these hormones in relation to major depressive episode but found no significant associations in the menopause transition [56].

In contrast, depressive symptoms adjusted for history of depression, race, and age were significantly associated with subject aggregate profiles of increased estradiol levels in the menopause transition in the POAS cohort (OR 1.27; 95 % CI: 1.00 – 1.60) [51]. In a subsequent longitudinal study of women with no history of depression, significant hormone associations with the onset of depressed mood included levels of follicle-stimulating hormone (FSH), decreased levels of inhibin b, decreased levels of luteinizing hormone (LH), and increased variability of estradiol, FSH and LH around the woman’s own mean level of each hormone [19]. Among women who met clinical diagnostic criteria for depressive disorder, increased levels of FSH and LH, decreased levels of inhibin b, and increased variability of estradiol and FSH were significant associated with the first occurrence of depression in the menopause transition (estradiol variability: OR 2.45; 95 % CI: 1.54 – 3.89). Possibly differences in the frequency and timing of hormone measures contributed to differing results between these studies.

Findings differed in other community-based studies. In the Melbourne study, a 2-year decline in estradiol levels was the strongest risk factor for depressive symptoms, adjusted for baseline depression, age and BMI (OR 3.41; 95 % CI: 1.24 – 9.36) [67]. While the findings suggested a role for declining estradiol in depression, the participants were postmenopausal, aged 56–67 years, and the assessments did not describe hormone associations with depression in the menopause transition. In contrast, the SMWHS found no significant association of urinary measures of estrone, FSH, testosterone and cortisol with depressed mood [33, 59]. A community-based, cross-sectional study of women ages 45–54 years found that 25 % experienced depressed mood (CES-D > =16), but there were no significant associations of depressed mood with estradiol or androgens [68].

The association between depressive symptoms and androgens of both adrenal and ovarian origin has also been studied. Two cross-sectional studies showed an inverse relationship between serum levels of dihydroepiandrosterone sulfate (DHEAS) and depression [69, 70], while longitudinal studies found either no significant association [55] or a positive association between DHEAS levels and depressive symptoms during the menopause transition [71].

The association between testosterone levels and depressive symptoms across the menopause transition is also controversial. In the 10-year SMWHS, there was no association between urinary testosterone levels and depressive symptoms [59]. However, in the early SWAN cohort, where the women were premenopausal or in the early menopause transition, there was a significant inverse association between testosterone and high depressive symptoms [72]. The association reversed in an 8-year follow-up, where testosterone levels were positively associated with depressive symptoms [55]. Further evidence from the Multiethnic Study of Atherosclerosis (MeSA) showed a significant inverse relationship between free testosterone levels and high depressive symptoms among women who were in the first 10 years postmenopause [73].

While these studies suggest possible associations of hormone fluctuations or changes with depression in the menopause transition, there are no definitive conclusions. It can be emphasized again that differences in the frequency and timing of hormone measurements, differences in measures of depressed mood, different comparisons of menopausal stages, and differing approaches and models in data analysis contribute to conflicting results.

### History of depression

A history of depression is one of the strongest predictors of depressive symptoms and depressive disorders in the menopause transition [18, 20, 51, 59, 74], with consistent findings in a number of large cohort studies.

An early report in the Massachusetts Women’s Health Study indicated that prior depression was the strongest predictor of subsequent depression (CES-D), with an odds ratio of 9.62 (95 % CI: 6.78– 13.70) [43]. In the Harvard Study of Moods and Cycles, women with a history of depression had a 20 % increased rate of entering the menopause transition sooner than women with no history of depression [74]. In the SWAN mental health study, the risk of MDD was three times greater for women with a history of MDD compared to women with no history of MDD (OR 2.98; 95 % CI: 1.55 – 5.72) [56]. In a follow-up of this study, twice as many women with a history of major depression (MDD) developed MDD compared to women with no history of MDD (59 and 28 %, respectively) [20]. Women with a history of depression in the POAS cohort had a 13 times greater risk of depressive symptoms in the menopause transition compared to women with no history of depression (OR 13.62; 95 % CI: 7.20 – 25.80, *P* < 0.001) [51]. In a longitudinal study of the pattern of depressive symptoms around menopause, approximately 50 to 65 % of women with a history of depression had high CES-D scores in the years before the FMP compared to 10 to 30 % of women with no history of depression [18]. In another large community-based, prospective study, women with prior depression were two times more likely to report increased depressive symptoms in the transition from pre- to perimenopause (OR 1.98; 95 % CI: 1.47 – 2.67) [75].

### Depression and vasomotor symptoms

Vasomotor symptoms (hot flashes and/or night sweats) are the most frequently reported menopausal symptoms across all races/ethnicities [76]. Many studies confirm their prevalence, which peaks around menopause, when more than 70 % of women report experiencing this symptom [32, 77]. In spite of their common occurrence, the etiology remains poorly understood, with diverse hypotheses and many associations with other behavioral, psychological and physical factors. A leading hypothesis is that hot flashes are triggered centrally by noradrenergic activation in association with changes in estrogen, which is known to influence thermoregulatory functioning [78]. While it is known that vasomotor symptoms are associated with changes in estradiol and other reproductive hormones, including follicle-stimulating hormone (FSH) and inhibin b, the associations are complex and do not clearly explain their occurrence [79, 80].

Although vasomotor symptoms (VMS) are highly correlated with depressive symptoms, evidence for the causal direction is conflicting. There are many differences in study designs, sample sizes and assessments of both VMS and depression, as delineated in an extensive review of the directional associations between VMS and depressive symptoms or major depressive disorder (MDD) [81]. The review included 17 cross-sectional studies that examined associations between VMS and depressive *symptoms* and 7 studies that examined the association between VMS and major depressive disorder (MDD); another 10 studies assessed the risk of women with VMS developing depressive symptoms, and 2 studies examined the reverse direction, i.e., the risk of women with high depressive symptoms developing VMS.

A statistically significant, positive association between VMS and depressive symptoms was identified in 9 of 17 cross-sectional studies, with odds ratios ranging from 1.27 (95 % CI: 1.08 – 1.51) to 8.1 (95 % CI: 2.5 – 26.4) [82, 83]. The researchers concluded that the evidence consistently supported *a positive association between VMS and depressive symptoms*, but the direction of association remained unclear. Of the 7 studies that examined the association between VMS and major depression, only 2 studies reported a significant association [63, 81], while 5 studies found no statistically significant association. However, the researchers determined that most of the 7 studies had moderate to high risk of bias due to small samples, convenience sampling and missing information, which strongly limited conclusions about the association between VMS and major depression.

Ten studies evaluated the risk of women with VMS developing depressive symptoms, and 8 of these indicated that VMS increased the risk of developing depressive symptoms, with odds ratios ranging from 1.62 (95 % CI: 1.43 – 1.84) to 8.88 (95 % CI: 2.57 – 30.68) [55, 84]. These studies were determined to be methodologically sound with adjustments for numerous covariates and low risk of bias, consequently providing consistent evidence for VMS increasing the risk of depressive symptoms. Further evidence was reported in a SWAN study, which investigated a very narrow time interval to show that VMS predicted next-day negative mood (OR 1.27; 95 % CI: 1.03 – 1.58); however, negative mood did not predict next-day VMS [85].

Two studies evaluated the risk of women with high depressive symptoms developing VMS, and both reported a positive association to suggest that depressive symptoms were more likely to precede hot flashes in women with no previous experience of either symptom [86, 79]. Women with consistently high depressive symptoms in the SMWHS were more likely to experience subsequent VMS [86]. Of women who had no high depressive symptoms or hot flashes at baseline in the POAS, 24 % reported depressive symptoms (high CES-D scores) before reporting hot flashes in a 10-year follow-up [79]. In contrast, only 8 % of the women reported hot flashes before reporting depressed mood.

Aims of this review did not include intervention studies, although they are clearly important for evaluating changes in hormone levels and associations of these changes with depressed mood and VMS. For example, estradiol administered to women with a diagnosis of depressive disorder in the menopause transition showed that estradiol improved depression independent of the presence of hot flashes [87]. In another study, estradiol was administered to women who had depressive disorders, hot flashes and disturbed sleep in the menopause transition. Results indicated that increased estradiol levels and improved sleep quality predicted improvement in depressed mood, while reduction of hot flashes did not improve depressed mood [88]. This evidence suggested that in spite of high correlations between depressive symptoms and VMS, these variables may have distinct pathways, and hot flashes alone are not a likely cause of depression around menopause.

These findings overall indicate that depressive symptoms and vasomotor symptoms frequently co-occur around the menopause transition. Endocrine events are a shared component of these symptoms but are not the only factor in their development. Numerous studies suggest that women with VMS have an increased risk of developing depressive symptoms, but the reverse direction is also observed. Data further indicate that menopausal depression can occur in the absence of VMS and that the cause of depression is not likely due to VMS alone. The studies suggest that VMS and depressive symptoms may have different underlying pathways and/or are modulated by different exogenous or endogenous factors. Further studies that are designed to unravel components of these complex associations are needed.

### Depression and poor sleep

Sleep difficulties are a common problem in the menopause transition. About 30 % of adults have one or more symptoms of insomnia, according to the American Academy of Sleep Medicine [89], and these symptoms are more prevalent in women, particularly in mid-life [90–93]. While a high prevalence of poor sleep quality in mid-life women is well-documented, several well-designed studies concluded that influences of both age and menopausal status on poor sleep were modest [92, 93]. In addition, while there is considerable evidence that sleep difficulties are correlated with depressive symptoms, poor sleep is a core symptom of clinical depression [94, 95], which frequently makes it difficult to determine whether sleep difficulties are a component of or independent of depressed mood or clinical depression.

The causal direction of poor sleep and depressed mood is particularly difficult to disentangle, in part due to the chronic nature of sleep problems, the episodic nature of depression and the many definitions of both disorders. The long-held domino hypothesis, i.e., that the association between sleep problems and depressed mood was driven by vasomotor symptoms has only partial support. For example, sleep problems predicted next-day negative mood, but an association with vasomotor symptoms was found only in women who had depression at the study baseline [96]. In an intervention study of women who had depressive disorders, increasing levels of estradiol and improved sleep quality predicted improvement in depressed mood in perimenopausal women, but improved hot flashes did not improve depressed mood [88]. The researchers concluded that changes in estradiol and sleep quality, rather than hot flashes, may mediate depression during the menopause transition.

A recent study of the association of poor sleep, mood and reproductive status found a significant association between depressive symptoms and sleep impairment in peri- and postmenopausal women but no consistent association in younger premenopausal women [97].

Reports from the SWAN cohort indicated that 31 % of the participants reported at least one of three evaluated sleep difficulties and identified significant associations between sleep difficulties and progression through the menopause transition [98].

In contrast, other studies found no significant association between depressed mood and poor sleep in perimenopausal women in adjusted analysis [99, 100]. In the study of Cheng et al. [100], anxiety was related to all assessed sleep problems but may have obscured an association with depressed mood due to high correlation of these variables. In an evaluation of poor sleep in relation to the final menstrual period, *premenopausal* sleep status was the strongest predictor of poor sleep in the menopause transition, and there was no further increase in poor sleep around the final menstrual period [101]. Again, methodological differences may contribute to conflicting findings, as for example, in the latter study, where most participants were not clinically depressed and poor sleep was reported at a premenopausal baseline. Measures of depression and anxiety were highly correlated, and anxiety rather than depression had the stronger association with poor sleep in these studies.

In summary, the findings show a high prevalence of sleep difficulties in mid-life women, but associations between increased risk of poor sleep in relation to menopause are complex with conflicting reports at this time. Depressive symptoms and sleep difficulties are common, the symptoms are correlated and both may increase around menopause. The causal directions and associations between these variables and the hormonal changes of ovarian aging as well as other psychosocial factors remain unclear.

### Other factors associated with menopausal depression

Numerous studies have identified significant associations of health, psychosocial and demographic variables with depressed mood around menopause, but evidence for the extent to which these factors are confounding or independently associated with depressed mood or clinical depression in perimenopausal women is inconsistent or lacking. Associations with depressive symptoms around menopause have been reported for health problems [54, 102], anxiety [57, 103], poor sleep and vasomotor symptoms [49, 79, 104–106], behavioral factors such as smoking and obesity [51, 54, 107], stress and negative life events [49, 53, 54, 57, 63, 107, 108], physical activity [109, 110], marital and relationship issues [59, 102], financial problems [54, 59], and demographic variables including race/ethnicity and education [19, 51, 54, 58]. Overall, the data support the possibility that depressed mood in the menopause transition is multifactorial and not simply due to menopausal status alone. It is possible that psychosocial and lifestyle factors, together with health experience, have more effect on depressed mood than endocrine changes. Although endocrine changes may be a trigger, the causal pathways remain unclear [31, 102].

Another important question that remains open is whether a first-onset depression in the menopause transition is etiologically or qualitatively different from recurrent depression. Several studies evaluated risk factors for a first-onset depression in the menopause transition for women with no history of depression. The risk of a first depression was significantly greater for women who reported vasomotor symptoms and for women who had a history of adverse life events in the Harvard Study of Moods and Cycles [36]. Vasomotor symptoms, high BMI and smoking were independent contributors to first onset of depressive symptoms in the POAS study [19]. Vasomotor symptoms at a trend level, prior health conditions and negative perceptions of functioning were risk factors for first onset of major depression in midlife women in the SWAN mental health study [20]. Steinberg et al. compared women with first-onset versus recurrent clinical depression and found that depressive symptom scores and measures of FSH did not differ between the two groups [111]. In the SWAN mental health study, the only risk factor uniquely associated with onset of major depression among women with no history of major depression was vasomotor symptoms (OR 2.09; 95 % CI: 1.26 – 3.47) [20].

Again, methodological differences may contribute to disparate findings. Different risk factors were evaluated and comparisons of menopausal status were not consistent across the studies. Furthermore, the risk factors identified for first-onset depression were also identified in studies that included women with recurrent depression, thus limiting conclusions. At present, there is no definitive evidence of distinct risks of a first-onset depression versus recurrent depression around menopause.

### Limitations and future directions

The majority of studies of depressed mood in the menopause transition reviewed here evaluated depressive symptoms, which are important for screening depression in the population and in clinical settings. Depressive symptoms are highly correlated with clinical diagnosis of depression but are not equivalent. Some studies evaluated samples with a diagnosis of depression, but reports of association between clinical depression and menopausal status are conflicting, and further studies are needed to determine whether the risk of diagnosed depression is increased around menopause. Studies of depression cross many domains, and this review did not include intervention or neuroendocrine studies, or in-depth consideration of the many of the identified psychosocial, behavioral and physical health factors that are important for understanding depression in midlife women. Other reviews are needed to address these areas. The reviewed studies were from different cohorts and population samples, and conclusions should be considered cautiously, particularly in comparing studies that differ in racial and social economic distributions, as recently demonstrated by Harlow et al. [64].

The greatest challenges for further research are to identify endocrine and genetic elements that may underlie depression and disentangle psychosocial factors that are associated with this disorder. Epidemiologic and clinical questions for further study include identifying the pattern and associated risk factors of depression following the final menstrual period; increasing information about associations between reproductive hormone changes and mood; further clarifying effects of hormone treatments on mood in perimenopausal women; determining confounding factors and causal directions of vasomotor symptoms and sleep difficulties in relation to depression and the extent to which these variables are associated with hormonal changes of menopause; disentangling depression and its associations with other major health problems of mid-life women such as cardiovascular disease, metabolic syndrome and osteoporosis. Longitudinal studies that clearly define menopausal stages, hormone measurements that are adequate for the questions and repeated measures of standard mood and behavioral assessments are important for increased understanding of women’s health in relation to menopause.

## Conclusions

While the changing hormonal milieu is experienced by all women around menopause, only a minority experience debilitating depressive symptoms in this transition period. Accumulating evidence indicates that depressive symptoms, and possibly clinical depression, increase in the menopause transition compared to premenopause. There is also evidence that the changing endocrine milieu is associated with increased depressive symptoms, but hormones are clearly not the only factor. Further studies are needed to understand the development of depression in women around menopause.

## Abbreviations

CES-D: Center for Epidemiologic Studies - Depression
DSM-IV: Diagnostic and Statistical Manual - IV
FMP: Final menstrual period
BMI: Body mass index
HADS: Hospital Anxiety and Depression Scale
HPA: Hypothalamic-pituitary-adrenal
OR: Odds ratio
CI: Confidence interval
RR: Relative risk
FSH: Follicle stimulating hormone
LH: Luteinizing hormone
MDD: Major depressive disorder
NIH: National Institutes of Health
STRAW: Stages of Reproductive Aging Workshop
SWAN: Study of Women’s Health Across the Nation
POAS: Penn Ovarian Aging Study
SMWHS: Seattle Midlife Women’s Health Study
